# Concentration-Governed Transition in DOM Function: From Surface Reductant to Performance Barrier on FeMnOx for Optimal Cr(VI) Removal

**DOI:** 10.3390/toxics14030231

**Published:** 2026-03-08

**Authors:** Yuxi Tang, Xiaole Ti, Rui Yang, Zeyu Zhang, Wenjie Zhang, Xiaojie Sun, Bin Dong, Ningjie Li

**Affiliations:** 1 Guangxi Key Laboratory of Environmental Pollution Control Theory and Technology, Guilin University of Technology, Guilin 541004, China; 2120240625@glut.edu.cn (Y.T.); 15040176160@163.com (R.Y.); 2010053@glut.edu.cn (W.Z.); 2 University Engineering Research Center of Watershed Protection and Green Development, Guangxi, Guilin University of Technology, Guilin 541004, China; 19287735235@163.com (X.T.); liciz2@163.com (Z.Z.); sunxiaojie@glut.edu.cn (X.S.); 3 Key Laboratory of Carbon Emission and Pollutant Collaborative Control, Education Department of Guangxi Zhuang Autonomous Region, Guilin University of Technology, Guilin 541004, China; tj_dongbin@163.com

**Keywords:** iron–manganese oxides, dissolved organic matter, chromium, reduction

## Abstract

Loading dissolved organic matter (DOM) onto iron–manganese oxides (FeMnOx) was a promising strategy for enhancing the hexavalent chromium (Cr(VI)) removal from wastewater. To optimize this process and gain deeper mechanistic insight, this study systematically investigated the DOM loading characteristics onto FeMnOx and its subsequent effect on Cr(VI) adsorption. DOM loading onto FeMnOx was significantly affected by the initial concentration of DOM and pH, with optimal loading conditions identified as a DOM concentration of 75 mg/L, pH of 4, ionic strength of 0.005 mol/L, temperature of 50 °C, and contact time of 4 h. During loading, FeMnOx preferentially adsorbed low-molecular-weight/low-aromaticity components such as tryptophan-like (C1) and fulvic acid-like (C2) substances. The adsorption process followed a non-uniform monolayer surface adsorption and involved multiple stages dominated by chemical interactions. DOM coating on FeMnOx significantly enhanced the Cr(VI) removal, and the maximum adsorption capacity under optimal loading conditions increased from 18.46 mg/g to 23.26 mg/g. Characterization by SEM-EDS, BET, ICP-MS, XPS, FTIR, and CV revealed that a moderate DOM loading (55–75 mg/L) enhanced the material’s surface reducibility and mesoporous structure. This improvement was attributed to the reduction of surface Mn(IV) to more-reactive Mn(III) by reductive functional groups in DOM, thereby promoting Cr(VI) adsorption and reduction. In contrast, excessive DOM loading (105 mg/L) formed a dense organic layer that masked active sites and hindered electron transfer, ultimately compromising the long-term reductive capability. These findings elucidate the concentration-dependent regulatory role of DOM in modifying FeMnOx properties, providing a theoretical foundation for the rational design of efficient DOM–metal oxide composites for heavy metal remediation in aquatic environments.

## 1. Introduction

Wastewater discharged from industries such as electroplating, leather tanning, metallurgy, and pigment manufacturing often contains high concentrations of hexavalent chromium (Cr(VI)). This pollutant is characterized by high toxicity, strong carcinogenicity, and a propensity for migration and diffusion in aquatic environments. It can penetrate biofilm, causing irreversible damage to DNA and proteins, thereby posing a serious threat to ecological safety and human health [1]. Therefore, the exploration of efficient remediation technologies for Cr(VI)-contaminated wastewater is of paramount importance for environmental protection and public health assurance.

Metal oxides, including iron oxides, manganese oxides, calcium oxides, and alumina, are recognized as effective adsorbents for heavy metal wastewater treatment [2]. Extensive research has demonstrated that composite bimetallic or trimetallic oxides exhibit superior adsorption performance compared to single-metal oxides [3,4]. Among these, iron–manganese oxides (FeMnOx) have garnered significant attention for heavy metal remediation due to their unique physicochemical properties. Characterized by a low zero-point charge, these materials maintain a negatively charged surface under typical environmental conditions [5], facilitating enhanced interactions with cationic pollutants. Furthermore, their redox-active surfaces enable concurrent adsorption and detoxification of hexavalent chromium (Cr(VI)) via reduction to the less-toxic trivalent form (Cr(III)). Given these synergistic functionalities, FeMnOx are promising choices for Cr(VI) wastewater treatment.

A series of methodologies have been identified to optimize the heavy metal adsorption performance of iron–manganese oxides, including adjusting the Fe/Mn molar ratio, pH, temperature, and ionic strength, etc. [5,6]. In addition, the surface modification strategy was often adopted to improve the adsorption of heavy metals by metal oxides. DOM has abundant functional groups, such as phenols, ketones, quinones, carboxyl groups, formyl groups, and aldehydes, facilitating its rapid adsorption onto the surfaces of nanoparticles, thereby altering the surface properties of the nanoparticles, for instance, by preventing their aggregation [7,8]. Our previous study also confirmed that loading DOM onto FeMnOx could promote the simultaneous adsorption and reduction of Cr(VI) [9], indicating it to be a strategy worthy of further study.

Wang et al. demonstrated that the loading degree of DOM on the adsorbent significantly influenced its adsorption performance [10]. Specifically, it was found that a thin coating layer of DOM on the four types of TiO_2_ nanoparticles resulted in the most pronounced enhancement of phenanthrene sorption. Too high a loading rate was very likely to lead to a decrease in the adsorption performance, and the diffusion process within the particles would be reduced due to the physical barrier effect. Environmental factors are likely to influence the loading process of DOM on metal oxides, including pH, ionic strength and temperature [11,12]. For example, the adsorption of DOM by magnetite is mainly dominated by electrostatic attraction at acidic pH, while the adsorption force may be dominated by coordination exchange when pH increases to 9, and the increasing ionic strength enhanced humic acid (HA) adsorption at each pH [13]. DOM concentration and pH played an important role during the electron transfer process of DOM [14]. The enhanced adsorption of DOM on magnetite at increased ionic strength was also reported [15]. It is necessary to optimize the loading degree of DOM on FeMnOx for better Cr(VI) adsorption.

The complex components of DOM, including protein-like substances, fulvic acid-like substances and humus-like substances, will undergo fractionation during the loading process of DOM on metal oxides [16]. Differences in the DOM fractions on metal oxides are also likely to affect the subsequent reduction of heavy metals. DOM directly acts as an electron shuttle, influencing the electron transfer between iron minerals and heavy metals such as Cr and arsenic. Low-molecular-weight HA exhibited higher reduction efficiency for Cr(VI), attributed to its rich polarity and aromatic structure, which provided abundant reaction sites for Cr(VI) adsorption, leading to its reduction [17]. The phenolic hydroxyl group in DOM was recognized as the main contributor to the redox of Cr(VI) during its adsorption by FeMnOx [9]. The aromatic DOM fractions with high-molecular-weights were preferentially adsorbed by the iron-based nanoparticles, i.e., nano zero-valent iron, Fe_2_O_3_, and Fe_3_O_4_ [8], similar to the results reported by Chekli et al. [18], while different from those reported by Illés et al. [15] that Fe_3_O_4_ preferred to adsorb humic acids with smaller molecular weight. Li et al. [19] demonstrated that the fulvic acid-like compounds of DOM were the main fractionated compounds on the surface of ferrihydrite. Even poorer fractionation selectivity on DOM by manganese oxide compared to iron oxide was found [20]. The phenolic compounds seemed preferentially oxidized by manganese oxide, reflecting the variability in reactivity of DOM’s composition [21,22]. The oxidized species of DOM had higher molecular weights and aromaticity [20], whereas Fe oxides would hinder the oxidation of DOM by Mn oxides [23]. DOM adsorbed by manganese oxide was oxidized more thoroughly at a lower pH, accompanied by more manganese reduction [20]. However, the DOM fractionation on FeMnOx under different conditions remain unclear.

Overall, loading DOM onto FeMnOx was a promising strategy for enhancing the Cr(VI) removal from wastewater, whereas the loading process of DOM onto FeMnOx and the ensuing Cr(VI) adsorption under different conditions were still unknown. This work presents a comprehensive investigation into the loading mechanisms of DOM onto FeMnOx and the subsequent concentration-dependent enhancement of Cr(VI) removal. The loading degree and fractionation of DOM on FeMnOx at different initial DOM concentrations, pH, ionic strength, and temperature were systematically analyzed through single-factor experiments and orthogonal experimental design. Subsequently, the Cr(VI) adsorption performance of FeMnOx loaded with DOM under different conditions was further investigated. Furthermore, multiple characterization techniques (SEM-EDS, ICP-MS, XPS, and CV) were employed to reveal the underlying mechanism. This study could not only provide references for the design and application of FeMnOx-DOM composites in wastewater treatment, but also for critical insights into the geochemical processes controlling organic carbon sequestration in environments dominated by FeMnOx [24].

## 2. Materials and Methods

### 2.1. Loading of DOM onto FeMnOx

FeMnOx was prepared by coprecipitation using potassium permanganate and ferrous sulfate at an Fe/Mn molar ratio of 3:1 under alkaline conditions (pH > 10), following established methodologies [9,25]. DOM loading experiments were performed as follows: 0.1 g FeMnOx was dispersed into 20 mL of DOM solution (prepared using humic acid, purchased from Macklin Biochemical Technology Co., Ltd., Shanghai, China.) with an initial concentration of A mg/L. The solution pH was adjusted to B, and ionic strength was maintained at C mol/L using NaCl. The mixture was ultrasonicated for 10 min, followed by continuous agitation at 180 rpm for E hours at a controlled temperature of D °C. After 10 min of settling, the suspension was centrifuged at 10,000 rpm for 20 min using a high-speed centrifuge. The resulting precipitate was labeled as FeMnOx-DOM. The loading process diagram is shown in Figure S1.

The supernatant was filtered through a 0.45 μm membrane and analyzed for DOM physicochemical properties. The loading efficiency of DOM onto FeMnOx was evaluated by measuring the total organic carbon (TOC) content of the DOM solution before and after loading. Leaching of iron and manganese during the loading process was quantified via inductively coupled plasma mass spectrometry (ICP-MS, NexION350X PerkinElmer, Waltham, MA, USA).

#### 2.1.1. Range of Each Factor in Single-Factor Tests

Group 1 (Initial DOM Concentration): A varied from 15 to 105 mg/L; B = 6; C = 0 mol/L; D = 30 °C; E = 4 h.

Group 2 (Solution pH): B varied from 2 to 10; A = 75 mg/L; C = 0 mol/L; D = 30 °C; E = 4 h.

Group 3 (Ionic Strength): C varied from 0 to 0.05 mol/L; A = 75 mg/L; B = 4; D = 30 °C; E = 4 h.

Group 4 (Temperature): D varied from 20 to 60 °C; A = 75 mg/L; B = 4; C = 0.005 mol/L; E = 4 h.

Group 5 (Contact Time): E varied from 0.5 to 24 h; A = 75 mg/L; B = 4; C = 0.005 mol/L; D = 50 °C.

#### 2.1.2. Orthogonal Test Design

Based on the results of single-factor experiments, each factor was optimized through orthogonal tests. The experiments were designed and analyzed by the software Design Expert, version 11.0 (State-Ease Inc., Minneapolis, MN, USA), and the specific level settings are shown in Table S1.

### 2.2. Physicochemical Characterization of DOM

#### 2.2.1. DOM Concentration Determination

Prior to analysis, the pH of DOM solutions before and after FeMnOx loading was adjusted to 4–5, followed by filtration through a 0.45 μm filtration membrane. TOC content was quantified using a TOC analyzer (Multi N/C 3100, Jena Analytical Instrument Co, Jena, Thuringia, Germany), with DOM concentration expressed as TOC. For UV-Vis and three-dimensional fluorescence (3D-EEM) spectroscopy, DOM solutions were diluted with ultrapure water to standardize TOC concentrations (7.5–33.4 mg/L), using ultrapure water as a blank.

#### 2.2.2. UV-Vis Spectroscopy

UV-Vis spectra (190–700 nm) were acquired using a UV5800PC spectrophotometer (Shanghai, China). Specific UV absorbance indices were calculated as follows: SUVA_254_ = A_254_/TOC, SUVA_280_ = A_280_/TOC, E_250_/E_365_ = A_250_/A_365_, and E_253_/E_203_ = A_253_/A_203_ [16]. The integrated absorbance (A_240–400_) within 240–400 nm was determined to assess molecular condensation and humification [26]. SUVA_254_, SUVA_280_, and E_250_/E_365_ were used to evaluate aromaticity and molecular weight, while E_253_/E_203_ reflected aromatic ring substitution patterns.

#### 2.2.3. 3D-EEM Spectroscopy

Fluorescence spectra were recorded on an F98 spectrometer (Lengguang, Shanghai, China) under the following conditions: 150 W xenon lamp, PMT voltage = 700 V, excitation (Ex) = 200–450 nm, emission (Em) = 280–550 nm, slit widths = 5 nm (Ex/Em), and scan speed = 3000 nm/min. Ultrapure water served as the blank. Parallel factor analysis (PARAFAC) via the DOMFluor toolbox decomposed the 3D-EEM data into components, with Fmax values representing relative fluorescence intensities [16].

### 2.3. Adsorption Model Fitting

To investigate the DOM-loading mechanism, the experimental data were fitted using common adsorption models. The equilibrium data were fitted using the Langmuir and Freundlich isotherm models. The kinetic data were fitted with the pseudo-first-order, pseudo-second-order, Elovich, and intraparticle diffusion models.

### 2.4. Characterization of FeMnOx and FeMnOx-DOM

The adsorbents loaded with varying concentrations of DOM were dried at 80 °C, ground, and prepared for characterization. After sputter-coating with gold for 30 s, the surface morphology and elemental composition of FeMnOx and FeMnOx-DOM were characterized using scanning electron microscopy (SEM; JSM-6380LV, JEOL, Akishima, Tokyo, Japan) coupled with energy-dispersive X-ray spectroscopy (EDS; IE350, Oxford Instruments, Abingdon, Oxfordshire, UK). The elemental composition and chemical species of C, Fe, Mn and Cr were analyzed by XPS (ESCALAB250Xi, Thermo Scientific, Waltham, MA, USA). The resulting high-resolution spectra were deconvoluted using the Avantage software (Version 5.9931, Thermo Fisher Scientific, Waltham, MA, USA) to identify the respective chemical states.

For FTIR measurement, a small amount of each sample was thoroughly mixed with dried spectroscopic-grade KBr powder under an infrared lamp using an agate mortar to minimize moisture interference. The mixture was pressed into transparent pellets, and spectra were recorded at a resolution of 4 cm^−1^ over 64 accumulated scans. The specific surface area was determined by nitrogen adsorption–desorption measurements at 77 K using an automated physisorption analyzer (ASAP 2460 M, Micromeritics, Norcross, GA, USA) and calculated by the Brunauer–Emmett–Teller (BET) method.

Cyclic voltammetry (CV) measurements were conducted to determine the redox potentials of the FeMnOx and FeMnOx-DOM composites. Specifically, 5 mg of FeMnOx loaded with different concentrations of DOM was transferred into a 5 mL centrifuge tube. A uniform ink was prepared by adding 280 μL of anhydrous ethanol and 20 μL of Nafion dispersion (DuPont, Wilmington, DE, USA), followed by ultrasonication for 30 min. Subsequently, 20 μL aliquots of the suspension were drop-cast onto fluorine-doped tin oxide (FTO) glass substrates and air-dried to fabricate the working electrodes. The measurements were performed using a three-electrode system on an electrochemical workstation (CHI 660E, CH Instruments, Shanghai, China). The system consisted of the prepared electrode as the working electrode, a Pt wire as the counter electrode, and an Ag/AgCl electrode as the reference electrode, with 0.5 M Na_2_SO_4_ aqueous solution as the electrolyte.

### 2.5. Batch Adsorption Tests of Cr(VI)

The simulated wastewater of Cr(VI) was prepared with potassium dichromate. Adsorption experiments were carried out in 50 mL conical bottles filled with 20 mL of Cr(VI) solution, and with the addition of adsorbents, shaking at 180 rpm. Post-adsorption, supernatants were centrifuged (8000 rpm, 10 min), filtered (0.45 μm), and analyzed for Cr concentration via ultraviolet and visible spectrophotometer in the method diphenyl carbazide spectrophotometry GB 7467-87 [27]. Triplicate runs were performed for each group. Cr(VI) adsorption by FeMnOx and FeMnOx-DOM were determined in the following conditions:

1. Effect of initial DOM concentration. Adsorption was conducted using 1 g/L of FeMnOx loaded with varying DOM concentrations (0–105 mg/L) in a 50 mg/L Cr(VI) solution at 40 °C and pH 8 for 4 h.
2. Effect of initial Cr(VI) concentration. Adsorption was performed with 1 g/L of FeMnOx-(75)DOM in Cr(VI) solutions ranging from 10 to 300 mg/L (natural pH 4.1–4.7) at 25 °C for 4 h.
3. Effect of adsorbents’ dosage. Adsorption tests used FeMnOx-(75)DOM at dosages of 0.5–7.5 g/L in 50 mg/L Cr(VI) solution at 25 °C for 4 h.
4. Effect of pH. The initial pH was adjusted to 3.0–10.0 using 5 g/L FeMnOx-(75)DOM at 25 °C for 4 h. In other tests, solution pH was not adjusted.
5. Effect of temperature. Adsorption was studied at 25–50 °C with 1 g/L FeMnOx-(75)DOM in 50 mg/L Cr(VI) solution for 4 h.
6. Effect of time. Adsorption kinetics were examined over 0.5–24 h using 1 g/L FeMnOx-(75)DOM in 50 mg/L Cr(VI) solution at 40 °C.

## 3. Results and Discussion

### 3.1. DOM Loading Behavior onto FeMnOx

#### 3.1.1. Key Factors Affecting Loading Efficiency

**Figure 1:**
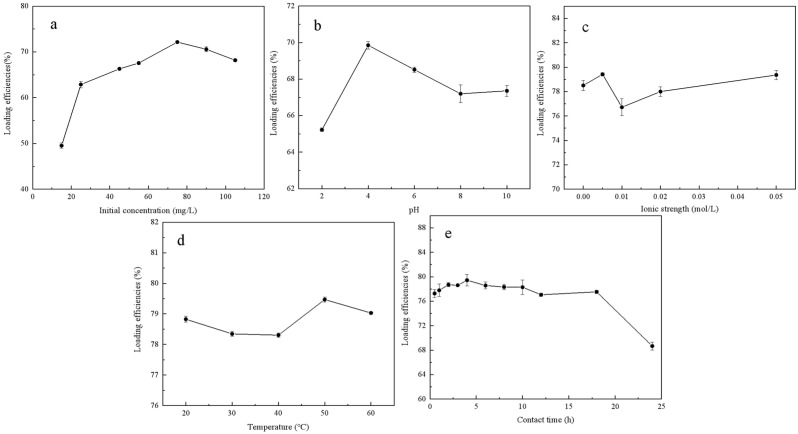
Loading efficiencies of DOM on FeMnOx under different conditions: (**a**) Initial concentration of DOM; (**b**) pH; (**c**) Ionic strength; (**d**) Temperature; (**e**) Contact time.

The loading efficiencies of DOM on FeMnOx under different conditions are shown in Figure 1. From Figure 1a, it was observed that as the initial concentration of DOM rose to 75 mg/L, the efficiency of loading DOM onto FeMnOx increased steadily from 49.5% to 72.1%. However, as the initial DOM concentration further increased to 105 mg/L, the loading efficiency decreased to 68.2%. The results indicate that while higher initial DOM concentrations initially resulted in more adsorption, the rate of loading DOM progressively decreased as saturation approached. Previous studies reported that an elevated concentration of DOM would enhance the sorption rate of small molecular DOM compounds onto iron oxide surfaces, leading to a higher loading rate, with larger molecular DOM species persisting in the solution [15].

The influence of pH values on loading efficiencies of DOM on FeMnOx is shown in Figure 1b. With an increase in pH from 2 to 4, the loading efficiency rose from 65.2% to a peak of 69.9%. Subsequently, as the pH surpassed 4, the loading efficiency gradually declined, stabilizing at approximately 67% within the pH range of 8–10. This trend aligns with the optimal pH range typically observed for DOM adsorption on magnetite [13]. Extremely low pH levels, such as pH 2, can lead to the dissolution of metal oxides, consequently hindering the adsorption of DOM. The decreased loading efficiency observed at pH values above 4 may be attributed to the increased negative charges associated with both DOM [12] and FeMnOx. Studies have indicated that manganese oxides may oxidize DOM more vigorously under acidic conditions [22]. To preclude the possibility that a decrease in dissolved organic carbon concentration was due to DOM mineralization, which would consequently skew the calculation of DOM loading, the experiment included direct measurement of TOC adsorbed onto FeMnOx (Method S1 and Figure S2). The results demonstrated a consistent trend with the loading efficiencies in Figure 1b, with the optimal loading observed at pH 4. This finding more definitively indicates that DOM loading was most favorable at a pH close to the isoelectric point of FeMnOx (i.e., 3.87) [9]. It confirms that the adsorption of DOM onto FeMnOx was a pH-dependent process, predominantly governed by electrostatic adsorption.

Figure 1c shows the effect of ionic strength on the loading efficiency of DOM on FeMnOx. Different ionic strengths have a negligible impact on the loading efficiency of DOM. Specifically, at an ionic strength of 0.005 mol/L, the DOM loading efficiency peaked at 79.4%. This phenomenon may be attributed to the prescribed experimental ion concentration range spanning from 5 to 50 mM. Previous studies suggest that variations in ionic strength influencing DOM adsorption typically range from 10 to 500 mM [15] or 4 to 309 mM [12], indicating that significant effects on DOM adsorption occur only when the ionic strength surpasses a certain threshold.

Figure 1d depicts that the loading efficiency of DOM marginally decreased with rising temperature before showing a gradual increase. The loading efficiency of DOM peaked at 50 °C, reaching a maximum of 79.5%, and subsequently declined beyond this threshold. Despite adsorption typically being influenced by temperature, the affinity and capacity of FeMnOx for DOM were not significantly affected within the range tested, aligning with previous findings by Engel [12].

Figure 1e illustrates the impact of contact time on the loading efficiency of DOM. The graph reveals that within 0.5 h of contact, the efficiency peaks at 77.3%, indicating rapid adsorption of DOM onto FeMnOx’s surface. At 4 h, the loading efficiency reached a maximum of 79.3%. Throughout an 18 h period, the loading efficiency remains stable between 77% and 80%. However, after 24 h of contact, the loading efficiency dropped to 68.6%. Prolonged contact time hindered the stable adsorption of DOM on FeMnOx, potentially triggering oxidation. Previous research notes a slight DOM oxidation within the initial 20 h of FeMnOx exposure to DOM, with a notable escalation in oxidation as contact time lengthened, resulting in reduced DOM molecular weight in the solution [20].

Based on the results of the single-factor experiments, it is evident that the loading process of DOM was significantly affected by the initial concentration of DOM and pH levels. The optimal loading conditions were at a DOM initial concentration of 75 mg/L, a pH of 4, an ionic strength of 0.005 mol/L (NaCl), a temperature of 50 °C, and a contact time of 4 h. A thorough investigation centered on the ideal values found in the single-factor analysis was carried out in order to further improve the loading circumstances. According to the results of the orthogonal experiments presented in Table S1, the optimal combination A_3_B_2_C_4_D_3_E_1_ aligned with the optimal loading conditions identified in the single-factor analysis.

#### 3.1.2. Selective Loading of DOM Components

**Figure 2:**
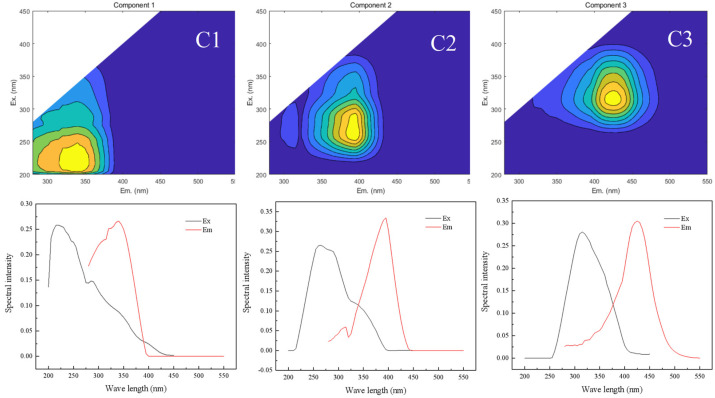
Components of DOM in PARAFAC analysis. C1, C2, and C3 represent trypsin-like compounds, fulvic acid-like compounds, and humic acid-like substances, respectively.

As illustrated in Figure 2, DOM was categorized into three components from PARAFAC analysis: C1 (Ex/Em = 230 nm/340 nm) denoting tryptophan-like compounds, C2 (Ex/Em = 250 nm/390 nm) representing fulvic acid-like compounds, and C3 (Ex/Em = 325 nm/425 nm) designating humic acid-like substances [16].

**Figure 3:**
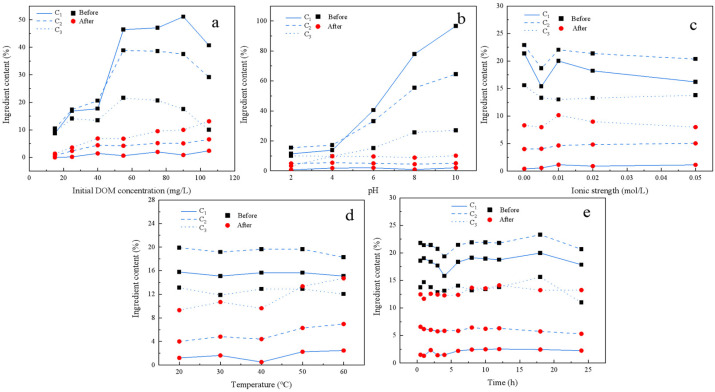
Changes in Fmax for each component of DOM before and after loading onto FeMnOx under different conditions. C1, C2, and C3 represent trypsin-like compounds, fulvic acid-like compounds, and humic acid-like substances, respectively. (**a**) Initial DOM concentration; (**b**) pH (**c**) Ionic strength; (**d**) Temperature; (**e**) Time.

The changes in Fmax for each component of DOM before and after loading onto FeMnOx under different conditions are depicted in Figure 3. The initial concentration of DOM and the pH value evidently affected the changes in fluorescence intensity of each component in the DOM stock solution, but the effects of temperature, oscillation time, and ionic strength were weak. Although within the studied range of DOM concentrations, the fluorescence intensity of each DOM component was not directly proportional to changes in DOM concentration (Figure 3a). This discrepancy was attributed to the fluorescence quenching effect induced by increased group concentrations, particularly evident when the initial DOM concentration exceeded 90 mg/L, resulting in a decrease in absorbance. Similar phenomena were also presented where Fmax of C3 increased after loading with FeMnOx at a reaction temperature of 60 °C (Figure 3d), or when the loading time was prolonged to 24 h (Figure 3e). Moreover, variations in pH influenced the Fmax of each component. With the pH range shifting from 4 to 10, the fluorescence intensity of all components increased, with C1 exhibiting the most significant enhancement. Allen also highlighted the impact of pH on the fluorescence properties of DOM, finding that the increase in pH value induced the expansion of the DOM structure, exposing more chromogenic base groups and resulting in an increase in its light absorption coefficient [28]. Nonetheless, we could still assess the relative content of each component after DOM loading onto FeMnOx, combined with the changes in TOC in the DOM solution before and after loading onto FeMnOx (Figure 2). Across all experimental conditions, the Fmax of the C3 component in the DOM solution was consistently lower than that of the C1 and C2 components. However, in the residual DOM after FeMnOx loading, the Fmax of the C3 component surpassed that of the C1 and C2 components. The Fmax alterations in C1 surpassed 83% in all tests, while the Fmax alterations in C2 varied between 60% and 93%, with minimal changes observed in the C3 component. These results indicate that FeMnOx was more likely to adsorb C1 and C2 in DOM. Notably, C1, a substance with a lower molecular weight than C3, demonstrated that FeMnOx tended to preferentially load more fulvic acid-like substances, similar to the DOM fractionation by ferrihydrite [19]. The reason probably lies in the presence of iron, mainly in the form of iron hydroxides, when FeMnOx was synthesized via the co-precipitation method in this study [29].

The original UV–Visible absorption spectra and the characteristic values of DOM before and after loading on FeMnOx are shown in Figure S3 and Table S2, respectively. In varied conditions, the SUVA_254_ and SUVA_280_ values of residual DOM after FeMnOx loading decreased, while the E_250_/E_365_ ratio increased. These results together indicate a lower molecular weight and aromaticity of residual DOM [26]. Additionally, the A_240–400_ value of DOM after FeMnOx loading decreased, suggesting a reduced level of molecular condensation and humification of DOM [30]. The UV–Visible absorbance of the original DOM with different concentrations varied evidently. At the same time, the difference became smaller when the other conditions, i.e., pH, temperature, ionic strength, and contact time, were changed under a fixed DOM concentration. Under optimal conditions, the SUVA_254_, SUVA_280_, and A_240–400_ values of residual DOM decreased by 87.65%, 89.49%, and 90.73% respectively, while the E_250_/E_365_ ratio increased by 66.98%. The E_253_/E_203_ value decreased from 0.5759 to 0.2559, indicating that the aromatic ring substitution groups of residual DOM predominantly comprised fatty chains that were less easily loaded onto FeMnOx [31]. Aromatic compounds with oxygen functional groups, on the other hand, were more readily adsorbed and coordinated with the FeMnOx surface through coordination processes. Previous studies also found that iron oxides preferentially adsorb aromatic compounds with high aromaticity in DOM, thereby increasing the proportion of aliphatic compounds in the solution [8].

#### 3.1.3. Adsorption Process

The adsorption equilibrium data for DOM onto FeMnOx were fitted using both the Langmuir [32] and Freundlich [33] isotherm models (Figure S4a,b). The Langmuir model exhibited a superior fit (higher R^2^ value) compared to the Freundlich model, indicating that the adsorption process is better described by homogeneous monolayer coverage on energetically uniform surface sites. This suggests that DOM molecules adsorb primarily as a single layer on the FeMnOx surface, with limited intermolecular interaction or multilayer formation. The maximum adsorption capacity derived from the Langmuir model was 75.39 mg/g. In contrast, the Freundlich isotherm, which assumes heterogeneous adsorption and multilayer formation, showed a lower goodness-of-fit. The Freundlich intensity parameter n was calculated to be 0.88 (where *n* = 1/b), falling within the range of 0.5–2.0, which corresponds to a moderately favorable adsorption process.

The adsorption kinetics of DOM on FeMnOx are presented in Figure S4c–f. High correlation coefficients (R^2^ > 0.99) were obtained for the pseudo-first-order [34], pseudo-second-order [35], and Elovich models [36], indicating a chemical adsorption process and surface energy heterogeneity. In contrast, the intraparticle diffusion model [37] exhibited a relatively low R^2^ value, and its fitted plot displayed a distinct intercept that did not pass through the origin. The existence of this intercept is usually related to the film or boundary-layer diffusion effect. The above results suggest that the adsorption of DOM on FeMnOx is a multi-step process governed by combined mechanisms. Specifically, the initial stage is likely controlled by film or boundary-layer diffusion, followed by a rate-limiting step dominated by surface chemical adsorption.

### 3.2. Impact of DOM Coating on Cr(VI) Removal

**Figure 4:**
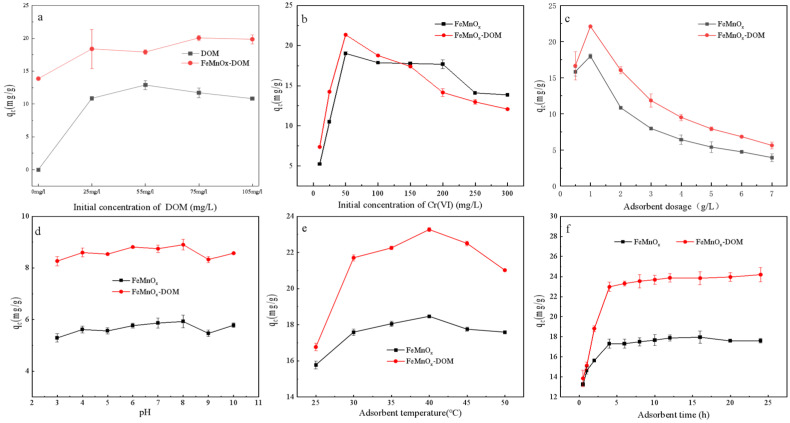
Influence of DOM coating on Cr(VI) adsorption by FeMnOx under different conditions: (**a**) Initial concentration of DOM; (**b**) Initial concentration of Cr(VI); (**c**) Adsorbent dosage; (**d**) pH; (**e**) Adsorbent temperature; (**f**) Adsorbent time.

The influence of DOM coating on the adsorption of Cr(VI) by FeMnOx under different conditions are shown in Figure 4.

As the initial DOM concentration increased from 0 to 105 mg/L, the adsorption capacity of Cr(VI) significantly increased. However, at lower DOM concentrations (e.g., 25 mg/L), its enhancement effect on Cr(VI) adsorption was not statistically significant. A discernible improvement in adsorption capacity manifested only when the initial DOM concentration reached or exceeded 55 mg/L. The maximum Cr(VI) adsorption capacity was achieved at an initial DOM concentration of 75 mg/L. Beyond this concentration, further increases in DOM loading resulted in a leveling off of the enhancement in Cr(VI) adsorption efficiency (Figure 4a). Consequently, to achieve a significant promotional effect of DOM on Cr(VI) adsorption, the recommended initial DOM concentration range is 55 to 75 mg/L.

As shown in Figure 4b–f, the optimal conditions for Cr(VI) adsorption by both adsorbents remained consistent at 1 g/L adsorbent, pH 8, temperature 40 °C, and 4 h of adsorption. Accordingly, FeMnOx-DOM exhibited a maximal adsorption capacity of 23.26 mg/g, surpassing FeMnOx at 18.46 mg/g under these optimal conditions and similar to the adsorption capacity of Fe-Mn oxides reported by Yang et al. [25]. The above results highlight the enhancement of FeMnOx’s Cr(VI) adsorption ability by DOM coating, influenced by contact time, temperature, and liquid/solid ratio, but minimally affected by pH. It demonstrates that the electrostatic attraction between the adsorbents and Cr(VI) was not the main force of Cr(VI) adsorption. Notably, this enhancement effect can be reversed to a weakening effect with changes in the liquid/solid ratio.

### 3.3. Surface Chemistry Evolution of FeMnOx and FeMnOx-DOM

Combined with the above results, it can be concluded that among the factors influencing the loading process of DOM onto FeMnOx and the removal of Cr(VI), the initial concentration of DOM has the most significant impact. To probe the evolution of FeMnOx’s surface chemistry in response to varying DOM loading concentrations, we characterized the samples using a suite of techniques, including BET, SEM-EDS, ICP-MS, XPS, CV, and FTIR.

The textural properties of the materials, particularly their pore structure and specific surface area, played a decisive role in determining the adsorption performance for Cr(VI). As shown in Figure S5, all samples exhibited Type IV isotherms with distinct H3-type hysteresis loops, indicative of mesoporous structures (2–50 nm) featuring slit-shaped or broadly distributed pore channels. Such an open and flattened pore architecture facilitated the diffusion of hydrated Cr(VI) ions (hydrated radius ≈ 0.3–0.5 nm) into the interior, reduced mass transfer resistance, and provided ample accessible surface for adsorption and subsequent reduction.

Quantitative analysis further supported this structural advantage. As summarized in Table 1 and Table S3, FeMnOx-(75)DOM possessed the highest BET specific surface area (298.56 m^2^/g) and the most developed microporous structure, reflected in its maximum micropore area and micropore volume. The abundance of micropores not only significantly increased the specific surface area but also ensured efficient contact between Cr(VI) ions and adsorption sites, given that the ion dimensions were well within the pore size range of all materials. Moreover, FeMnOx-(75)DOM exhibited the highest total pore volume (0.50 cm^3^/g), which further enhanced its capacity for ion accommodation and internal mass transfer. These structural merits were consistent with its superior maximum adsorption capacity derived from the Langmuir isotherm model, confirming that the enhanced surface accessibility and optimized pore geometry collectively contributed to the improved adsorption performance.

As shown in Figure S6, the loading of DOM onto FeMnOx resulted in the dissolution of both iron and manganese under all conditions, with manganese dissolution being predominant. Temperature, loading time, and initial DOM concentration had significant influences. Higher initial DOM concentrations led to greater manganese dissolution and less iron dissolution from the surface. Extreme pH (pH 2) notably enhanced iron dissolution, which was only pronounced at this pH. Nevertheless, substantial manganese dissolution did not impair the extent of DOM loading, consistent with previous studies, confirming that iron oxides are the primary components responsible for the adsorption of DOM in FeMnOx [19].

SEM analysis revealed that the surficial Fe/Mn ratios of FeMnOx or FeMnOx-DOM were consistently lower than the bulk Fe/Mn ratios determined by ICP-MS (Table 1), likely due to the enrichment of manganese oxides on the surface. The bulk Fe/Mn ratio of the unloaded iron–manganese oxides was higher than the theoretical value, possibly due to the loss of manganese oxides during sample preparation, which involved extensive rinsing with water to neutral pH. After DOM loading, the surface Fe/Mn ratio decreased further with increasing initial DOM concentration, indicating that the DOM loading process promoted additional surface enrichment of manganese oxides. This was consistent with the analysis results of SEM-EDS (Table 1 and Figure S7), with the Fe/Mn atomic ratio of original FeMnOx being approximately 3:1, while being obviously higher in FeMnOx-DOM prepared under the optimal loading condition. The co-precipitation effect induced by manganese ions would lead to the indiscriminate loading of all DOM components onto manganese oxide. This phenomenon was identified as the underlying reason for the inferior fractionation effect of manganese oxide compared to iron oxide [20]. However, this study revealed a distinct fractionation effect on DOM, reconfirming that iron oxide in FeMnOx likely played a predominant adsorption role rather than manganese oxide.

**Figure 5:**
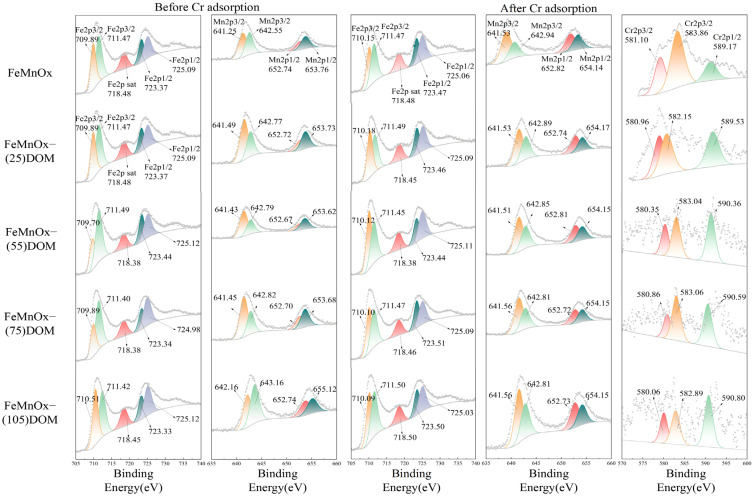
XPS spectra of FeMnOx and FeMnOx-DOM before and after Cr adsorption.

Figure S8 presents the XPS spectra of FeMnOx-DOM and FeMnOx-DOM-Cr. The deconvolution of the high-resolution Fe 2p, Mn 2p, and Cr 2p spectra, performed with Avantage software, is shown in Figure 5. Detailed parameters of XPS peak fitting are shown in Table S4. In the Fe 2p region, the spectra were fitted with multiple peaks corresponding to Fe 2p_3/2_ (706.5–711.5 eV), Fe 2p_1/2_ (722.0–725.1 eV), and associated satellite features (Fe 2p*sat, 714.0–720.0 eV). The spectral components with binding energies of 706.5–710.0 eV (for Fe 2p_3/2_) and 722.0–723.6 eV (for Fe 2p_1/2_) were assigned to Fe(II) species. The remaining peaks in the main doublets, along with the satellite peaks, were attributed to Fe(III) species [38]. The Mn 2p spectra were characterized by the Mn 2p_3/2_ (638–645 eV) and Mn 2p_1/2_ (649.9–655.0 eV) spin–orbit doublets. Based on previous studies, the deconvolution allowed for the identification of different manganese oxidation states: Mn(II) in MnO (640.1–640.9 eV for Mn 2p_3/2_ and 653.3–653.8 eV for Mn 2p_1/2_), Mn(III) in MnOOH (640.9–641.5 eV for Mn 2p_3/2_ and 651.8–653.3 eV for Mn 2p_1/2_), and Mn(IV) in MnO_2_ (641.5–645.0 eV for Mn 2p_3/2_ and 653.8–655.0 eV for Mn 2p_1/2_) [39]. The deconvolution of the Cr 2p XPS spectrum reveals two primary contributions: the peak at ~580 eV (Cr 2p_3/2_) is attributed to Cr(III) hydroxides, while the peak at ~590 eV (Cr 2p_1/2_) corresponds to Cr(VI) oxyanions [40]. The raw Cr 2p XPS spectra were smoothed using a Savitzky–Golay algorithm with a window of nine points (Origin) to reduce random noise. As shown in Figure 5, the signal-to-noise ratio of the Cr 2p XPS spectra progressively deteriorated with increasing initial DOM loading concentration in FeMnOx-DOM. This is probably attributed to the enhanced attenuation of Cr 2p photoelectrons by the thickening organic overlayer, which increases the probability of inelastic scattering during their escape from the surface. The relative abundance of each valence state was estimated by calculating the corresponding peak area ratio, with the specific results being presented in Table 1.

XPS analysis showed that as the initial DOM concentration increased from 0 to 75 mg/L, the proportion of Fe(II) on the surface slightly decreased from 39.60% to 30.88%, while the proportions of Mn(II) and Mn(III) increased from 19.21% to 20.31% and from 50.83% to 58.04%, respectively. Conversely, the proportion of Mn(IV) decreased from 29.96% to 21.65%. At a DOM concentration of 105 mg/L, the surface proportions of Fe(II), Mn(II), and Mn(III) decreased sharply to 12.34%, 15.50%, and 17.16%, respectively. Meanwhile, the proportions of Fe(III) and Mn(IV) increased to 87.64% and 67.34%, respectively. These observations suggest that within an appropriate DOM concentration range, the reducing character of DOM dominates, reducing surface Mn(IV) to Mn(III). However, at extremely high concentrations (105 mg/L), DOM molecules may rapidly form a dense organic coating on the surface, masking the reduced active iron and manganese species (i.e., Fe(II), Mn(II), Mn(III)). This coating could physically hinder further contact between internal Mn(IV) and the reducing functional groups of external DOM, thereby suppressing continued reduction. At this point, the complexation-promoted dissolution effect of DOM likely surpasses its reduction effect, becoming the dominant process.

After Cr adsorption, the surface Cr atomic ratios on FeMnOx-(0, 55, 75, 105)DOM-Cr were 0.07%, 0.49%, 0.51%, and 0.42%, respectively (Figure S9), indicating that the removed Cr was immobilized on the adsorbent surface, and these values parallel the adsorption efficiencies shown in Figure 4a. Post-adsorption of Cr(VI), the adsorbent surface contained only Fe(III), Mn(III), and Mn(IV). The proportion of Mn(IV) increased from 76.97% to 81.87% as the DOM concentration rose from 0 to 75 mg/L, while FeMnOx-(105)DOM-Cr showed a slightly lower Mn(IV) proportion of 80.45%. This suggests that the reducible species Fe(II) and Mn(II) were completely oxidized, and only part of Mn(III) participated in Cr(VI) reduction. In the 0–75 mg/L DOM groups, the proportion of surface reducible species correlated positively with Cr removal efficiency. As shown in Figure S10, the Cr(III)/Cr(VI) ratios in FeMnOx-(0, 25, 55, 75, 105)DOM-Cr were 0.24, 0.46, 0.71, 1.13, and 0.77, respectively. Notably, FeMnOx-(75)DOM-Cr exhibited the highest surface Cr(III)/Cr(VI) ratio, further confirming FeMnOx-(75)DOM’s superior performance in reducing Cr(VI) to Cr(III). Although the proportion of active species was lower in FeMnOx-(105)DOM, good removal efficiency of Cr was still observed, possibly due to compensatory surface complexation by hydroxyl and carboxyl groups in DOM. This aligns with our previous findings that surface complexation and redox reactions are the main mechanisms for Cr(VI) adsorption by FeMnOx and FeMnOx-DOM [9].

Residual reducible species, specifically Mn(III), remained on the adsorbent surface after Cr adsorption. This may be attributed to the alkaline conditions of the adsorption experiments, differing from our earlier acidic condition studies where manganese was fully oxidized to Mn(IV) [9], indicating faster Cr(VI) reduction rates under acidic conditions. It is well-documented that Cr(VI) reduction is strongly pH-dependent, with significantly higher efficiency under acidic conditions than in alkaline environments [41]. This appears to contradict the observation that the surface Cr(III)/Cr(VI) ratios under alkaline conditions (FeMnOx-Cr, 0.24; FeMnOx-(75)DOM-Cr, 1.13) were higher than those reported in our previous acidic condition study (FeMnOx-Cr, 0.08; FeMnOx-(75)DOM-Cr, 0.59) [9]. One possible explanation is that under alkaline conditions, Cr(III) directly precipitates and deposits on the adsorbent surface, potentially encapsulating or passivating active sites. In contrast, under acidic conditions, the reduced Cr(III) primarily exists as soluble Cr(OH)^2+^ species.

**Figure 6:**
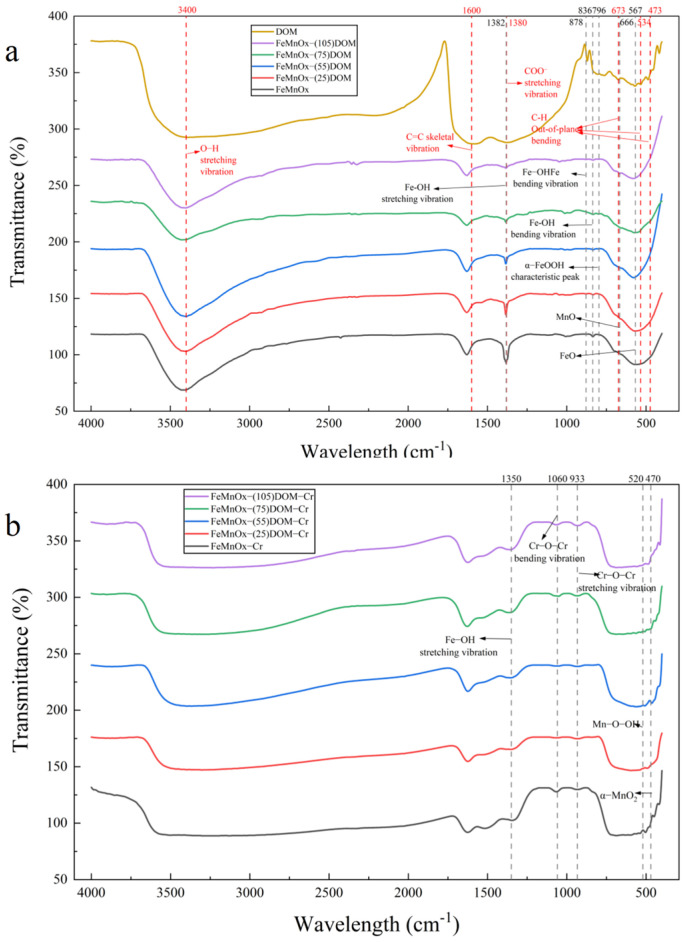
FTIR survey spectra: (**a**) DOM and FeMnOx-(0, 25, 55, 75, 105)DOM; (**b**) FeMnOx-(0, 25, 55, 75, 105)DOM-Cr.

Surface functional groups were analyzed using Fourier transform infrared (FTIR) spectroscopy (Figure 6). Characteristic peaks of DOM were observed at 3400 cm^−1^ (O–H stretching vibration), 1600 cm^−1^ (aromatic C=C skeletal vibration), 1380 cm^−1^ (COO^−^ stretching vibration), and at 673, 534, and 473 cm^−1^ (out-of-plane bending of aromatic C–H). For FeMnOx, distinct peaks were identified at 880 cm^−1^ (assigned to Fe–OH–Fe), 796 cm^−1^ (characteristic of α-FeOOH), and at 1382 cm^−1^ and 836 cm^−1^, corresponding to the bending and stretching vibrations of Fe–OH bonds, respectively. The characteristic peaks for Fe–O and Mn–O were observed at 567 cm^−1^ and 626 cm^−1^, respectively. These Fe–O and Mn–O functional groups have been widely reported to exhibit high reactivity, enabling them to interact with anionic pollutants and form complexes such as Fe–O–M and Mn–O–M.

Spectral comparison of DOM, FeMnOx and FeMnOx-DOM reveals that the peaks at 3400 cm^−1^, 1600 cm^−1^, and 673 cm^−1^ shift to varying degrees. The COO^−^ stretching vibration peak overlaps with the Fe–OH bending vibration peak, and the intensity of this combined peak gradually decreases with increasing DOM loading concentration. Furthermore, the characteristic peaks at 534 cm^−1^ and 473 cm^−1^ disappear. These observations confirm the successful loading of DOM onto the FeMnOx surface and indicate that the interaction pattern is influenced by the loading concentration.

Examination of the FTIR spectrum of FeMnOx before and after Cr(VI) adsorption shows that, aside from a shift in the Fe–OH bending vibration peak, the other characteristic Fe/Mn-related peaks largely disappear. New absorption peaks emerge, which are attributed to Mn–O–OH, α-MnO_2_, and Cr–O–Cr. Additionally, a broad and intense absorption band appears in the 500–700 cm^−1^ region, characteristic of Cr–O stretching vibrations. These spectral changes confirm the effective adsorption of Cr(VI) onto the material surface, thereby demonstrating the feasibility of this material for Cr(VI) removal.

**Figure 7:**
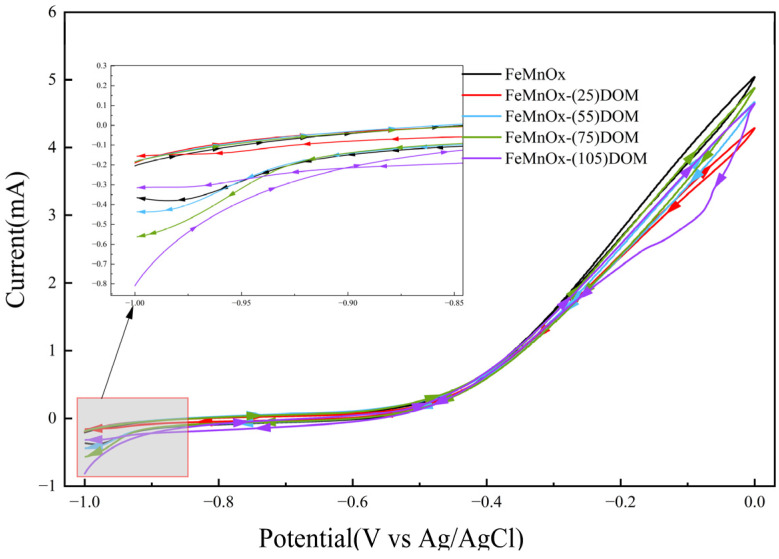
Cyclic voltammetry curves of different FeMnOx-DOM composites.

The CV curves of FeMnOx-(0-105)DOM all exhibited tilted spindle-like shapes without sharp symmetric peaks (Figure 7). Such a profile is indicative of pseudocapacitive behavior, commonly observed in metal oxides [42]. It also indicates that the reductive charge of both FeMnOx and FeMnOx-DOM primarily originated from surface and near-surface processes. A stable and gradually varying current response was observed across all adsorbents within the potential window of −0.5 to −1.0 V. As the initial DOM concentration increased from 0 to 105 mg/L, the average cathodic current densities for the five materials were 0.3656, 0.1562, 0.4375, 0.5642, and 0.3162 mA, respectively (Table 1). This suggests that the reduction current of FeMnOx-DOM improved only when the initial DOM concentration exceeded 25 mg/L, with FeMnOx-(75)DOM exhibiting the highest reduction current. This trend is consistent with the Cr(III)/Cr(VI) ratio obtained from XPS analysis. Furthermore, integration of the cathodic scan segments of the CV curves revealed that, except for FeMnOx-(25)DOM, the total cathodic charge of each adsorbent showed a positive correlation with the initial DOM concentration. These findings suggest that loading sufficient DOM could enhance the overall reducibility of the adsorbents, with FeMnOx-(105)DOM possessing the highest overall reduction capability. However, the sustained reduction rate of FeMnOx-(105)DOM was lower, likely due to reduced electron transfer efficiency at active sites, which may be caused by the masking of surface reductive active sites by DOM.

### 3.4. Concentration-Dependent Regulatory Mechanism for DOM

The integrated characterization results delineate a concentration-dependent mechanism where DOM’s role evolves from a reductant to an optimized interface layer, and finally to a physical/electronic barrier:

Low Concentration Range (<55 mg/L): DOM primarily acts as a reductant. Its reducing functional groups (e.g., phenolic -OH) convert surface Mn(IV) to more active Mn(III), enhancing reducibility without forming a dense layer.

Optimal Concentration Range (55–75 mg/L): DOM forms a performance-optimizing interface. It maximizes the surface concentration of reduced Mn species (Mn(II)/Mn(III)) and creates a microenvironment conducive to electron transfer, thereby achieving peak reduction current and Cr(VI) removal efficiency without significantly impeding mass transfer.

Overload Concentration (105 mg/L): DOM transforms into a physical barrier and electronic insulator. The thick, possibly disordered organic coating blocks contact between reactive sites and reactants, hinders electron transfer efficiency, and ultimately diminishes the sustained reduction rate despite a high total reducible charge.

This concentration-dependent transition underpins the observed optimal DOM loading for enhancing Cr(VI) removal by FeMnOx, highlighting the delicate balance between providing beneficial surface modification and causing obstructive site masking.

## 4. Conclusions

This study demonstrated that DOM loading onto FeMnOx was critically influenced by the initial DOM concentration and pH. The adsorption process followed a non-uniform monolayer surface adsorption and involved multiple stages dominated by chemical interactions. FeMnOx exhibited selective adsorption of DOM components, preferentially loading low-molecular-weight and low-aromaticity fractions. A moderate DOM coating (initial concentration of 55–75 mg/L) effectively modulated the surface properties of FeMnOx by reducing surface Mn(IV) to Mn(III), optimizing the pore structure, and increasing the number of active sites, thereby significantly enhancing the adsorption and reductive removal of Cr(VI). In contrast, excessive DOM loading formed a physical barrier that hindered reactant mass transfer and electron transfer, leading to diminished performance. Therefore, an optimal DOM loading concentration range exists, within which DOM functions as a beneficial interface-optimizing layer; beyond this range, it transforms into an inhibitory physical and electronic barrier. Under the optimal conditions, the maximum Cr(VI) adsorption capacity of FeMnOx-(75)DOM reached 23.26 mg/g, notably higher than that of unmodified FeMnOx (18.46 mg/g), and the FeMnOx-(75)DOM composite exhibited the highest surface Cr(III)/Cr(VI) ratio (1.33) after adsorption. These findings provide deeper insight into the interaction mechanisms between DOM and metal oxides and offer important process parameters and theoretical guidance for optimizing DOM–metal oxide composites for the efficient removal of Cr(VI) from water.

## Figures and Tables

**Table 1:** Surface chemical evolution results of FeMnOx and FeMnOx-DOM.

	SEM-EDS	ICP-MS	XPS												CV		BET
	Fe/Mn Molar Ratio		Before Cr(VI) Adsorption (%)					After Cr(VI) Adsorption (%)							Current (mA)	Total Reductive Charge (C)	Specific Surface Area (m^2^/g)
			Fe (II)	Fe (III)	Mn (II)	Mn (III)	Mn (IV)	Fe (II)	Fe (III)	Mn (II)	Mn (III)	Mn (IV)	Cr (III)	Cr (VI)			
FeMnOx	3.33	3.58	39.60	60.40	19.21	50.83	29.96	0.00	100	0.00	23.03	76.97	19.37	80.63	0.37	1.098 × 10^−4^	233.78
FeMnOx- (25)DOM	3.28	3.44	35.22	64.78	19.28	56.66	24.06	0.00	100	0.00	20.80	79.20	31.59	68.41	0.16	4.548 × 10^−5^	274.01
FeMnOx- (55)DOM	3.22	3.17	33.83	66.17	19.81	56.90	23.29	0.00	100	0.00	19.70	80.30	41.45	58.55	0.44	1.188 × 10^−4^	258.85
FeMnOx- (75)DOM	3.18	3.25	30.88	69.12	20.31	58.04	21.65	0.00	100	0.00	18.13	81.87	53.06	46.94	0.56	1.488 × 10^−4^	298.56
FeMnOx- (105)DOM	3.11	3.33	12.34	87.66	15.50	17.16	67.34	0.00	100	0.00	19.55	80.45	43.56	56.44	0.32	1.608 × 10^−4^	242.41

## Data Availability

Full disclosure of data: All data supporting this study have been made public in Web of Science. Please refer to the references section of the article for the data from the relevant datasets cited.

## Supplementary Materials

The following supporting information can be downloaded at: https://www.mdpi.com/article/10.3390/toxics14030231/s1, Method S1: Experimental design for the effect of loading pH on organic carbon content of FeMnOx-DOM; Table S1: Orthogonal experimental results; Table S2: The characteristic values of UV-Visible absorption spectra of DOM before and after loading on FeMnOx; Table S3: BET data of FeMnOx loaded with different concentrations of DOM; Table S4: Detail parameters of XPS peak fitting; Figure S1: Schematic illustration of DOM adsorption onto FeMnOx; Figure S2: Effect of loading pH on organic carbon content of FeMnOx-(55)DOM; Figure S3: The original UV-Visible absorption spectra of DOM before and after loading on FeMnOx; Figure S4: Analysis of Adsorption Models of DOM onto FeMnOx: (a,b) Adsorption isotherm Models; (c–f) Adsorption kineticsModels; Figure S5: BET adsorption desorption curves of different FeMnOx-DOM composites; Figure S6: Leaching of Fe and Mn from FeMnOx during DOM’s loading process; Figure S7: SEM-EDS images of FeMnOx and FeMnOx-DOM; Figure S8: XPS survey spectra: (a–e) FeMnOx-(0, 25, 55, 75, 105)DOM; (f–j) FeMnOx-(0, 25, 55, 75, 105)DOM-Cr; Figure S9: SEM-EDS images of FeMnOx-DOM-Cr; Figure S10: The correlation between DOM loading concentration and Cr(III)/Cr(VI) ratio.

## Abbreviations

The following abbreviations are used in this manuscript:

TOC	Total Organic Carbon
ICP-MS	Inductively Coupled Plasma Mass Spectrometry
SEM	Scanning Electron Microscope
EDS	Energy Dispersive Spectroscopy
XPS	X-ray Photoelectron Spectroscopy
FTIR	Fourier Transform Infrared Spectroscopy
CV	Cyclic Voltammetry Curve
BET	Brunauer–Emmet–Teller
