# Supplementary material for: Concentration-Governed Transition in DOM Function: From Surface Reductant to Performance Barrier on FeMnOx for Optimal Cr(VI) Removal
Source: Toxics. 2026 Mar 8;14(3):231. doi: 10.3390/toxics14030231 (PMC13030821; doi:10.3390/toxics14030231)
Supplement: Supplementary file 1 [file toxics-14-00231-s001.zip › toxics-4156673-supplementary.pdf]

Supplementary materials for

Concentration-governed transition in DOM function: from surface reductant to performance barrier on FeMnOx for optimal Cr(VI) removal

Yuxi Tang[a], Xiaole Ti[b], Rui Yang[a], Zeyu Zhang[b], Wenjie Zhang[a], Xiaojie Sun[b], Bin Dong[c], Ningjie Li[a,c,*]

[a] Guangxi Key Laboratory of Environmental Pollution Control Theory and Technology, Guilin University of Technology, Guilin 541004, China

[b] University Engineering Research Center of Watershed Protection and Green Development, Guangxi, Guilin University of Technology, Guilin 541004, China

[c] Key Laboratory of Carbon Emission and Pollutant Collaborative Control, Education Department of Guangxi Zhuang Autonomous Region, Guilin University of Technology, Guilin, 541004, China

[*] Corresponding author's e-mail address: echoningjie_333@126.com, liningjie@glut.edu.cn (Ningjie Li)

Method S1 Experimental design for the effect of loading pH on organic carbon content of FeMnOx-DOM.

The organic carbon contents of FeMnOx-DOM composites synthesized at various loading pH was quantified using a TOC analyzer (Multi N/C 3100, Jena Analytical Instrument Co, Germany). In this procedure, 50 mL of a 55 mg/L DOM solution was adjusted to initial pH values of 2-10, and transferred into 150 mL conical flasks. Then, 0.25 g of FeMnOx was added to each flask, and the mixtures were ultrasonicated for 10 min to ensure homogeneity. The suspensions were subsequently agitated at 50 °C and 180 r/min for 4 h in a shaking incubator. The solid products, designated as FeMnOx-(55)DOM, were then collected by centrifugation at 4000 r/min for 10 min. The resulting adsorbents were dried at 80 °C, treated with 10% HCl overnight to remove inorganic carbon, and finally dried again at 80 °C before TOC measurement. FeMnOx was used as a control. The loading efficiency of DOM was also calculated by dividing the organic carbon content of FeMnOx-(55)DOM by TOC in the solution before DOM loading.

Table S1 Orthogonal experimental results.

	A	B	C	D	E	Loading efficiency of DOM (%)
Test 1	55	3	0	40	4	69.60
Test 2	55	4	0.001	45	4.5	72.70
Test 3	55	5	0.003	50	5	72.90
Test 4	55	6	0.005	55	5.5	73.50
Test 5	65	3	0.001	50	5.5	75.40
Test 6	65	4	0	55	5	76.00
Test 7	65	5	0.005	40	4.5	72.80
Test 8	65	6	0.003	45	4	71.10
Test 9	75	3	0.003	55	4.5	73.00
Test 10	75	4	0.005	50	4	76.30
Test 11	75	5	0	45	5.5	73.20
Test 12	75	6	0.001	40	5	73.50
Test 13	85	3	0.005	45	5	73.90
Test 14	85	4	0.003	40	5.5	75.00
Test 15	85	5	0.001	55	4	75.10
Test 16	85	6	0	50	4.5	75.70
K_1	72.175	72.975	73.625	72.725	73.025	
K_2	73.825	75.000	74.175	72.725	73.550	
K_3	74.000	73.500	73.000	75.075	74.075	
K_4	74.925	73.400	74.125	74.400	74.275	
R	2.750	2.025	1.175	2.350	1.250	
Optimal combination				$A_3B_2C_4D_3E_1$		

Note: K represents the average value of the experimental results for each level; R represents the range, with a larger R value indicating a greater impact of the factor on the experimental results.

Table S2 The characteristic values of UV-Visible absorption spectra of DOM before and after loading on FeMnOx.

Group		Before					After				
		$SUVA_{254}$	$SUVA_{280}$	$A_{240\sim400}$	E_{250}/E_{365}	E_{253}/E_{203}	$SUVA_{254}$	$SUVA_{280}$	$A_{240\sim400}$	E_{250}/E_{365}	E_{253}/E_{203}
DOM Concentration (mg/L)	15	2.5676	2.2720	23.0540	2.6327	0.5155	1.3606	1.1284	10.8934	10.9397	0.1911
	25	4.5749	3.9705	44.4992	2.6946	0.6329	1.1189	0.9498	10.0373	11.4851	0.1714
	40	5.3939	4.7252	82.2720	2.4530	0.6961	0.7397	0.6116	9.9237	15.5467	0.1834
	55	6.4393	5.6294	126.6453	2.4500	0.6784	0.5378	0.4633	9.0086	21.7551	0.2288
	75	6.5832	5.8384	144.0766	2.4169	0.5867	0.4398	0.4000	8.7351	11.2697	0.1959
	90	6.8118	6.2050	199.7946	2.0780	0.4572	0.3570	0.2994	8.4328	23.3256	0.2302
	105	12.7876	12.1102	405.9843	3.5187	1.1079	0.2855	0.2455	9.0842	9.7075	0.1726
pH	2	4.8694	4.5905	164.7106	1.6462	0.7412	15.7829	14.9500	433.5939	9.2445	1.1093
	4	5.7916	5.4044	160.6890	1.8155	0.7172	0.4750	0.3992	10.3832	13.9545	0.2492
	6	7.8547	7.0392	180.8108	2.4313	0.4448	0.3825	0.3352	8.1328	17.0000	0.2379
	8	8.1698	7.4480	199.5373	2.3872	0.4837	0.2319	0.2257	5.7101	7.3636	0.1556
	10	8.6578	8.0859	221.5432	2.3486	0.5253	0.2225	0.2166	5.7101	7.3636	0.1556
Ionic strength (mol/L)	0	7.0556	6.7778	10.9322	1.4977	0.6764	0.7735	0.6731	10.9322	4.4883	0.2250
	0.005	6.7140	6.4767	11.6768	1.4350	0.6635	0.8500	0.7199	11.6768	4.8920	0.2301
	0.01	8.3961	8.0739	11.6515	1.6406	0.3305	0.7876	0.6843	11.6515	5.3729	0.2024
	0.05	6.0508	5.5784	13.0372	1.8197	0.7698	0.9382	0.7896	13.0372	3.8782	0.1859

Table S2 (continued) The characteristic values of UV-Visible absorption spectra of DOM before and after loading on FeMnOx.

Group		Before					After				
		$SUVA_{254}$	$SUVA_{280}$	$A_{240\sim400}$	E_{250}/E_{365}	E_{253}/E_{203}	$SUVA_{254}$	$SUVA_{280}$	$A_{240\sim400}$	E_{250}/E_{365}	E_{253}/E_{203}
Temperature (℃)	20	7.0789	6.6711	136.3416	1.7255	0.5346	0.6351	0.5455	9.7189	4.3682	0.2334
	30	6.9340	6.5858	139.5789	1.6572	0.5018	0.6907	0.5858	10.4142	5.2035	0.2493
	40	6.9772	6.6451	143.0124	1.6021	0.4214	0.7788	0.6506	11.9287	4.9368	0.2582
	50	6.8053	6.5085	132.2783	1.5813	0.5759	0.8405	0.6842	12.2593	4.7895	0.2559
	60	7.0574	6.7878	139.8511	1.5971	0.4669	0.8145	0.6431	12.0290	5.0037	0.2526
Time(h)	0.5	7.2161	6.9202	147.4473	1.5728	0.2942	0.5773	0.5127	9.6690	3.7362	0.2312
	1	7.2296	6.9371	142.3098	1.6280	0.2872	0.6667	0.5811	10.6150	4.2451	0.2454
	2	7.0947	6.7958	130.1881	1.6427	0.5186	0.8648	0.7221	12.7246	4.6931	0.2652
	3	7.3684	6.9832	138.5647	1.6326	0.3941	0.8586	0.7024	12.4366	4.9853	0.1410
	4	7.3200	7.0007	132.2783	1.5813	0.5759	0.8908	0.7192	12.6345	4.9380	0.2444
	6	7.2493	6.9368	135.1266	1.6133	0.4838	0.8810	0.7305	12.9474	5.1758	0.2759
	8	7.6506	7.5385	183.8332	1.6065	0.3548	0.7271	0.5940	12.3297	6.9749	0.2738
	10	7.5087	7.3148	177.6802	1.5771	0.3445	0.6952	0.5625	11.5679	16.4444	0.2734
	12	7.2976	6.9874	148.0564	1.6234	0.2992	0.7728	0.6321	11.6199	12.0182	0.2749
	18	7.2622	6.9635	142.4389	1.6302	0.2912	0.7782	0.6481	11.6235	9.0769	0.2786
	24	7.7476	7.6409	224.1872	1.5953	0.4333	0.5894	0.4915	12.4948	6.7537	0.2835

Table S3 BET data of FeMnOx loaded with different concentrations of DOM.

Group	Specific surface area (m²/g)			Volume (cm³/g)			Aperture (nm)	
	Langmuir	t-Plot	BJH	Total hole	t-Plot	BJH	BET	BJH
FeMnOx	1329.52	74.72	193.25	0.46	0.04	0.44	7.92	9.14
FeMnOx-(25)DOM	1245.46	113.35	226.68	0.48	0.06	0.45	6.95	7.97
FeMnOx-(55)DOM	1263.89	93.57	234.72	0.49	0.05	0.48	7.63	8.22
FeMnOx-(75)DOM	1308.52	132.66	228.66	0.50	0.07	0.46	6.69	8.12
FeMnOx-(105)DOM	1117.16	94.61	213.75	0.44	0.05	0.43	7.28	8.01

Table S4 Detail parameters of XPS peak fitting.

		Scan	FWHM FeMnOx	FeMnOx-(25)DOM	FeMnOx-(55)DOM	FeMnOx-(75)DOM	FeMnOx-(105)DOM
Before Cr(VI) adsorption	Fe	Scan1	1.81	1.76	1.85	1.79	1.82
		Scan2	2.18	2.18	2.15	2.19	2.20
		Scan3	2.37	2.38	2.37	2.37	2.36
		Scan4	1.83	1.78	1.79	1.82	1.88
		Scan5	2.46	2.40	2.49	2.46	2.45
	Mn	Scan1	1.97	1.9	1.83	1.83	1.84
		Scan2	1.75	1.68	1.77	1.64	2.05
		Scan3	2.16	2.08	2.05	2.02	2.39
		Scan4	2.02	1.94	1.92	1.92	2.38
After Cr(VI) adsorption	Fe	Scan1	1.85	1.83	1.87	1.86	1.82
		Scan2	2.17	2.20	2.15	2.15	2.19
		Scan3	2.38	2.37	2.35	2.38	2.40
		Scan4	1.88	1.87	1.90	1.83	1.83
		Scan5	2.44	2.46	2.40	2.39	2.41
	Mn	Scan1	2.04	2.02	2.02	2.02	2.02
		Scan2	2.14	2.13	2.11	2.11	2.12
		Scan3	1.97	1.92	2.04	1.92	2.05
		Scan4	2.06	2.02	2.02	2.02	2.05
	Cr	Scan1	2.12	2.12	2.13	2.13	2.13
		Scan2	2.45	2.46	2.44	2.46	2.45
		Scan3	2.38	2.4	2.38	2.4	2.39

Note: Scan peak is arranged from low to high binding energy. All peaks were fitted using XPSPEAK software with a Shirley-type background correction. The peak shapes were modeled using a Gaussian-Lorentzian mixed function, where the Lorentzian/Gaussian mixing ratio was set to 30%.

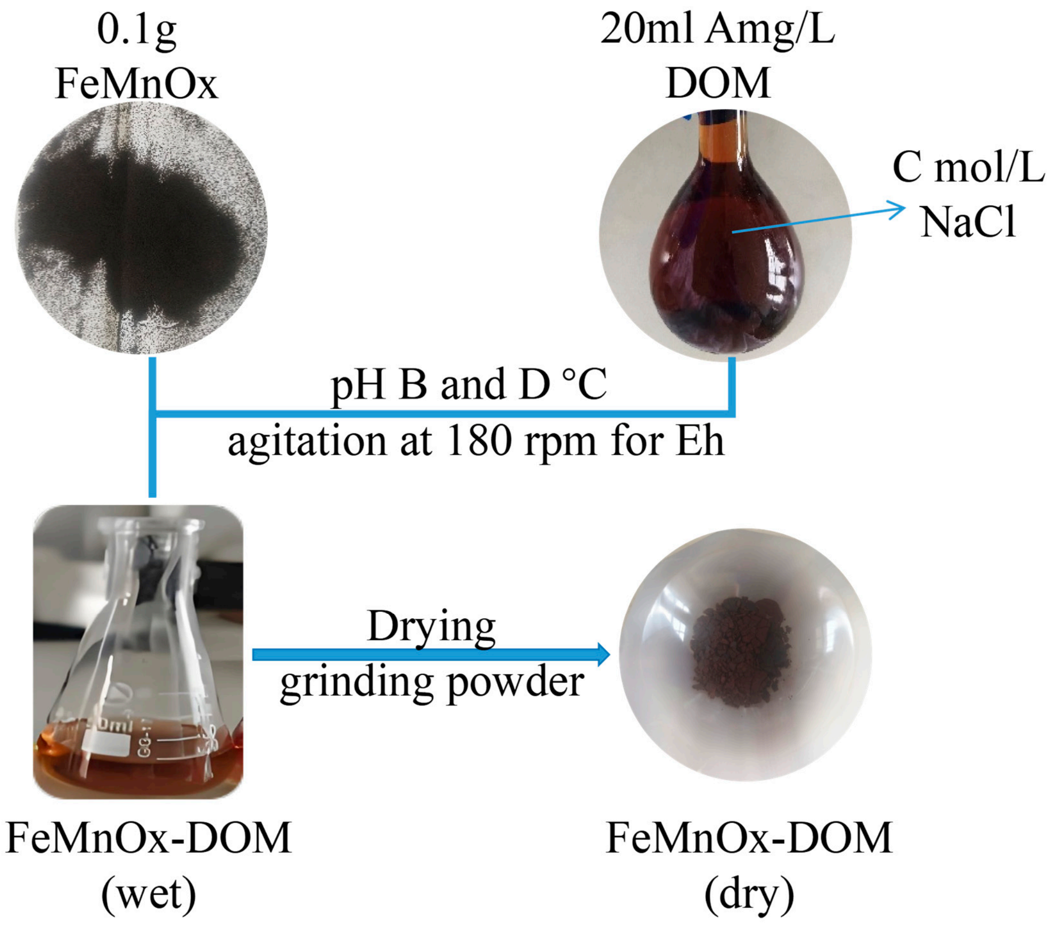

Figure S1 Schematic illustration of DOM adsorption onto FeMnOx.

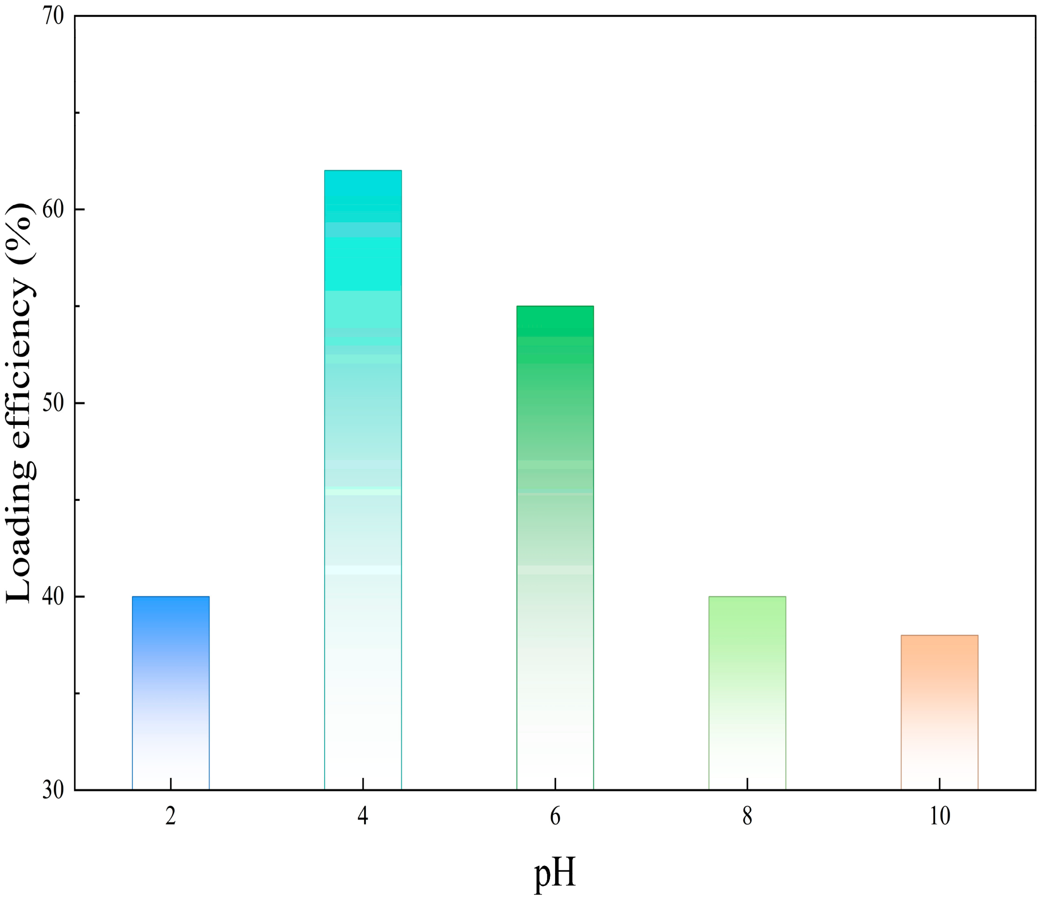

Figure S2 Effect of loading pH on organic carbon content of FeMnOx-(55)DOM.

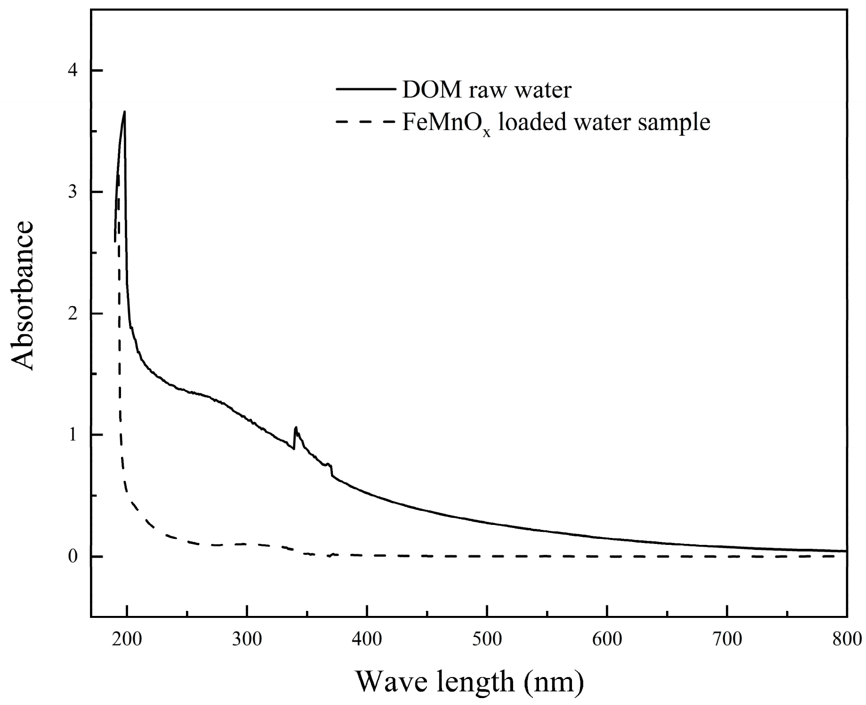

Figure S3 The original UV-Visible absorption spectra of DOM before and after loading on FeMnOx.

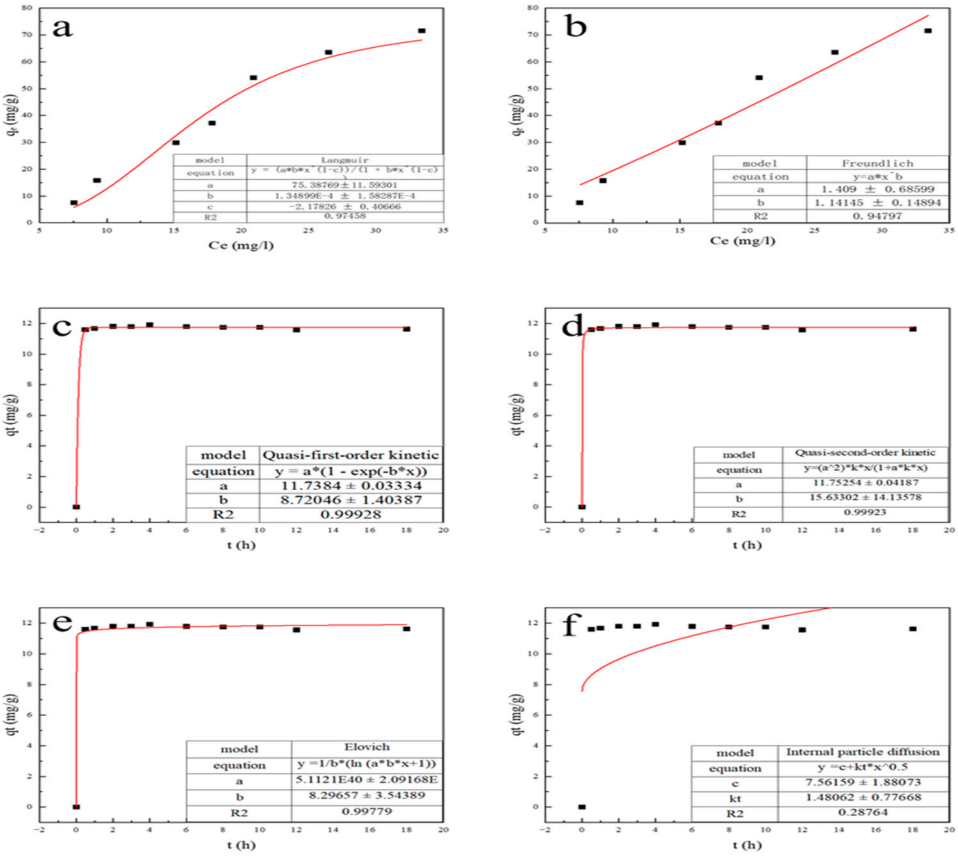

Figure S4 Analysis of Adsorption Models of DOM onto FeMnOx: (a-b) Adsorption isotherm Models; (c-f) Adsorption kineticsModels.

The corresponding formulas are:

Langmuir Model： $q_e = q_m * k_l * c_e / 1 + k_l * c_e$ (1)

Freundlich Model： $q_e = k * c_e \hat{} (-n)$ (2)

Quasi first order reaction kinetic model： $q_t = q_e * (1 - \exp(-k_1 * t))$ (3)

Quasi second order reaction kinetic model： $q_t = ((a_1 \hat{} 2) * k_2 * t / (1 + a_1 * k_2 * t)$ (4)

Elovich model： $q_t = 1/b * (\ln(a_2 * b * t + 1))$ (5)

Internal particle diffusion model： $q_t = c + k_t * t \hat{} 0.5$ (6)

In the formula: c_e - adsorption equilibrium concentration (mg/L);

q_e - equilibrium adsorption capacity (mg/g);

q_m - maximum adsorption capacity (mg/g);

k_l - Langmuir adsorption coefficient (L/mg);

k,n - Freundlich adsorption constant;

q_e - adsorption capacity at equilibrium (mg/g);

q_t - adsorption capacity at time t (mg/g);

k_1- Quasi first order reaction kinetic equation adsorption rate constant (min^{-1});

k_2- Quasi second order reaction kinetic equation adsorption rate constant (g/(mg · min));

a_1, a_2, b, c - Calculate constants;

k_t - diffusion rate constant within particles.

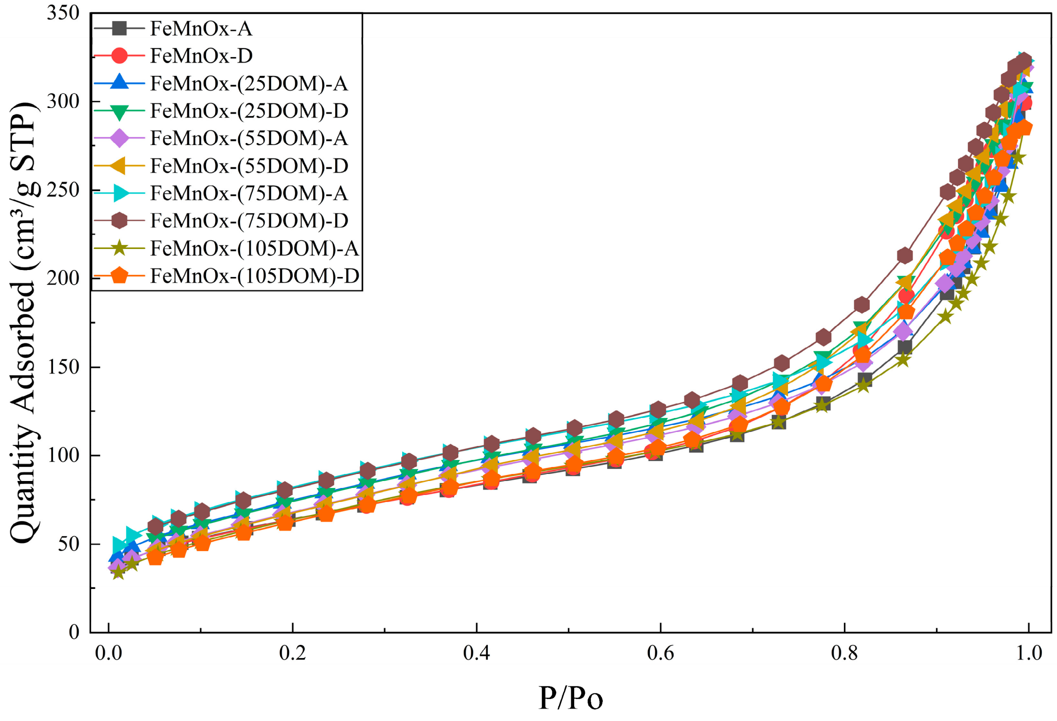

Figure S5 BET adsorption desorption curves of different FeMnOx-DOM composites.

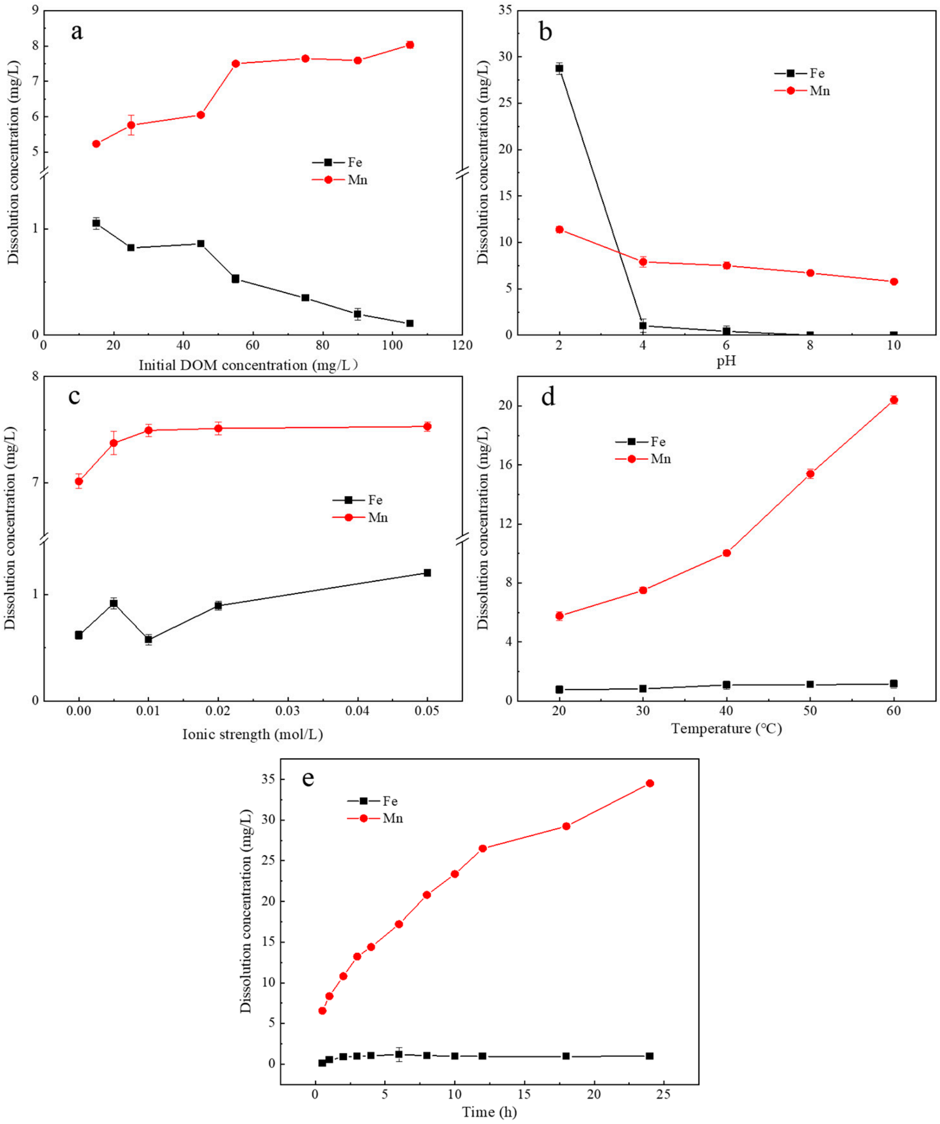

Figure S6 Leaching of Fe and Mn from FeMnOx during DOM's loading process.

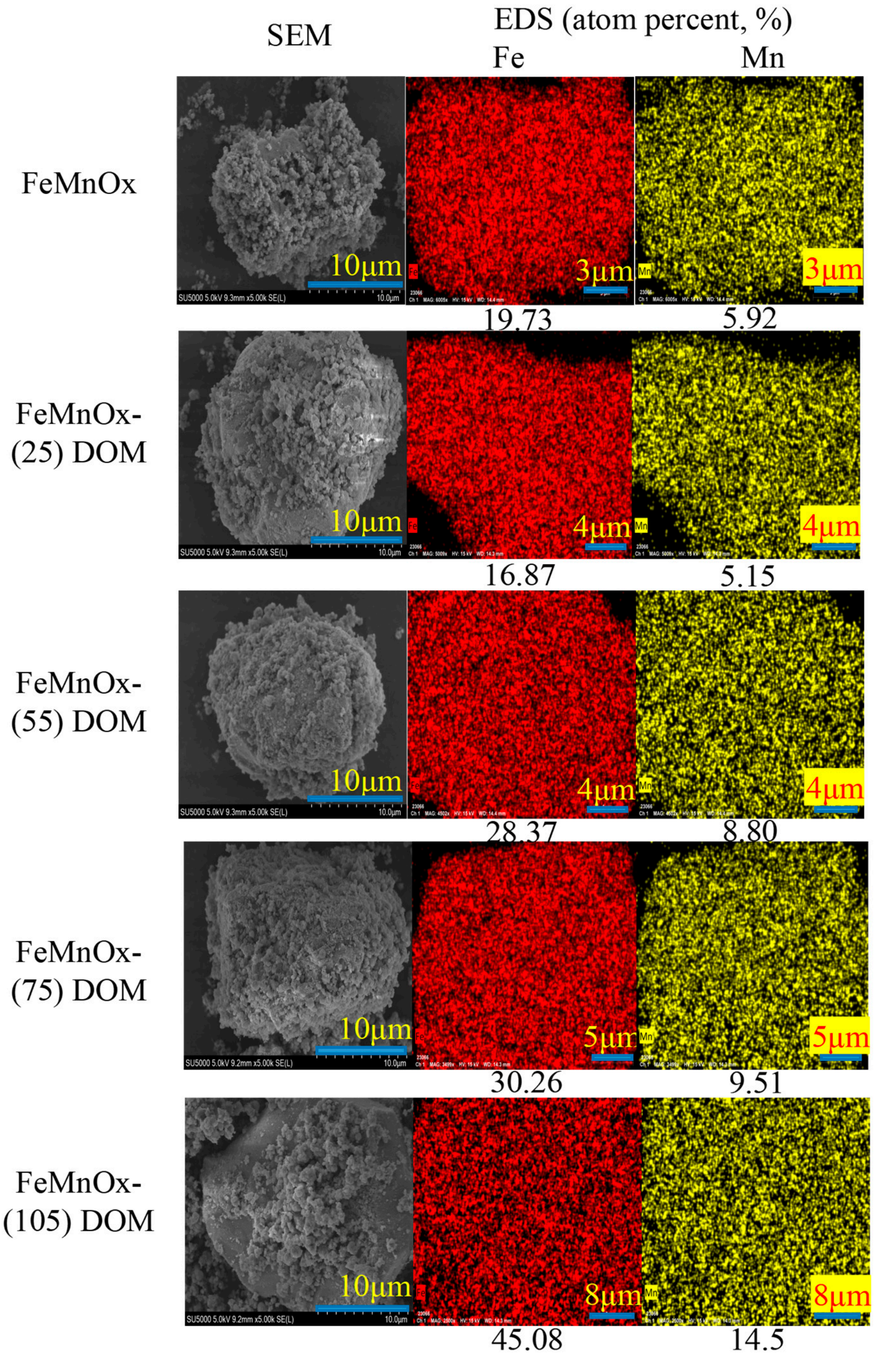

Figure S7 SEM-EDS images of FeMnOx and FeMnOx-DOM.

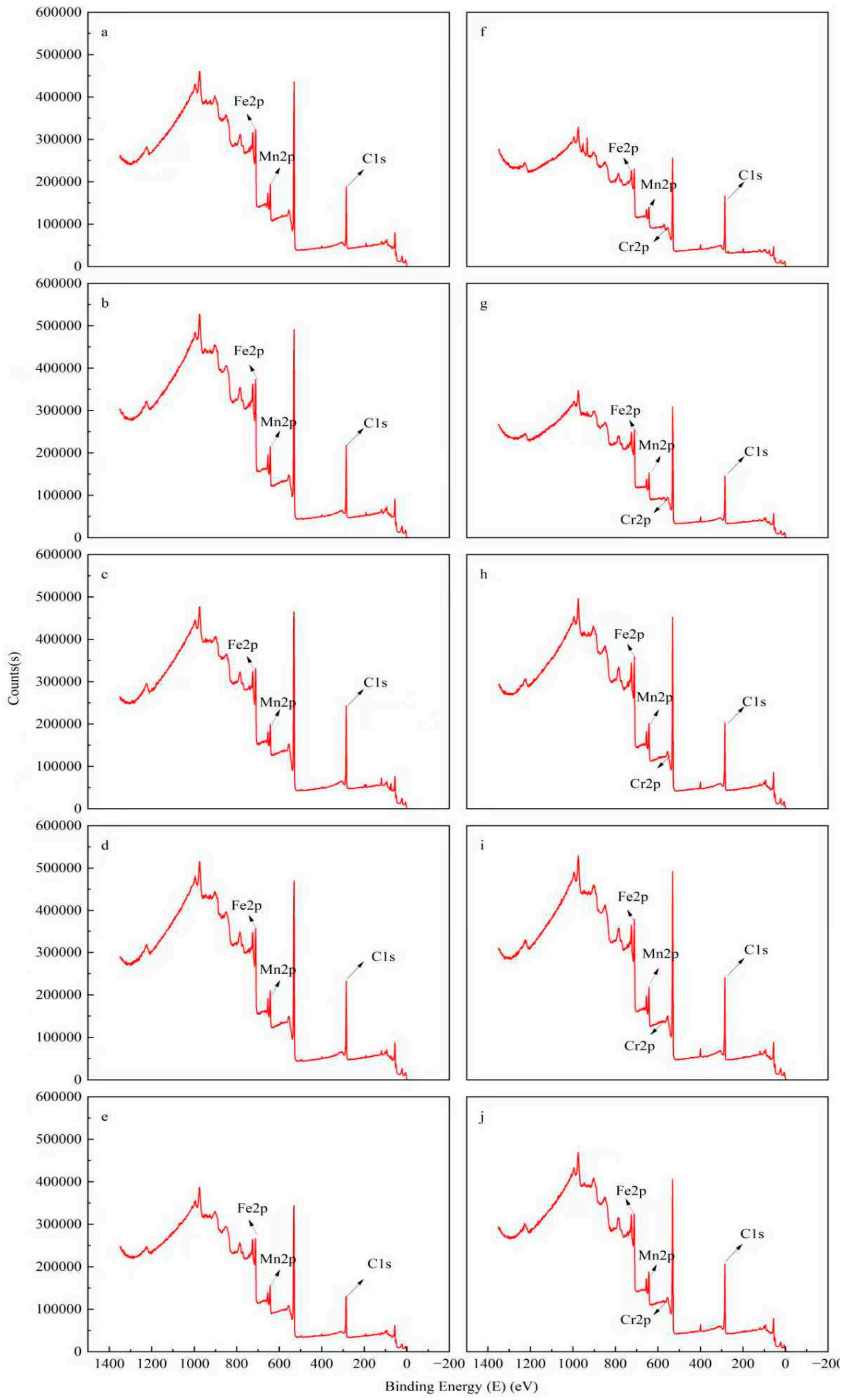

Figure S8 XPS survey spectra: (a-e) FeMnOx-(0, 25, 55, 75, 105)DOM; (f-j) FeMnOx-(0, 25, 55, 75, 105)DOM-Cr.

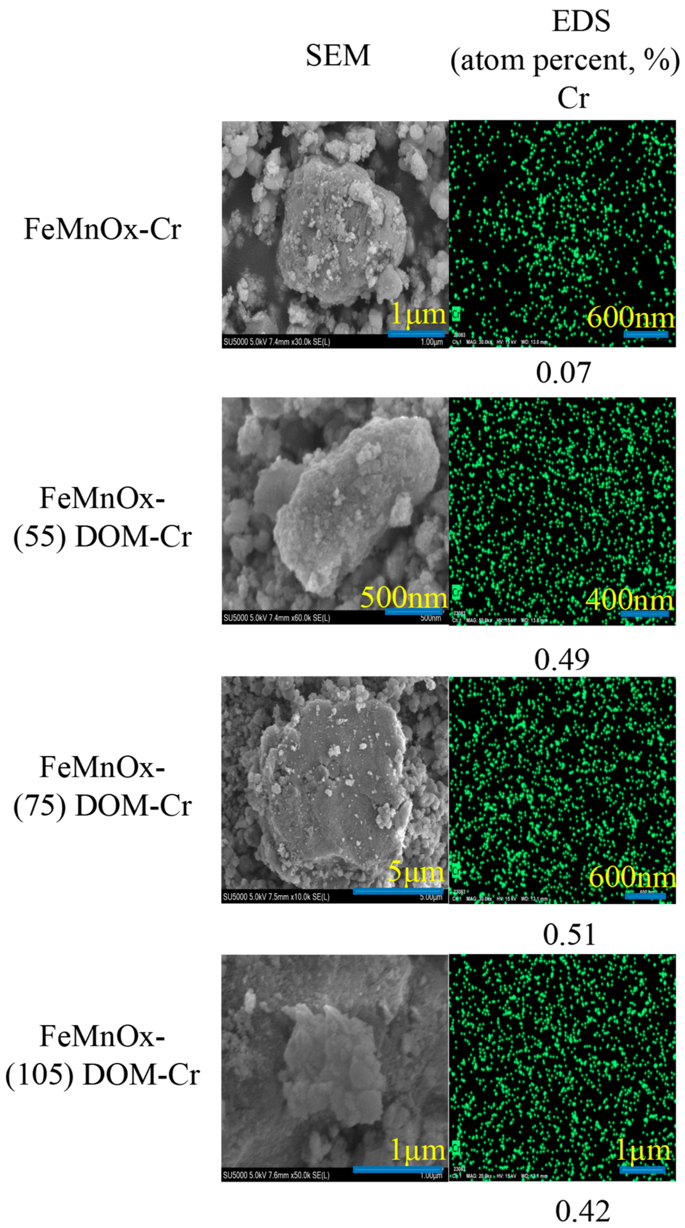

Figure S9 SEM-EDS images of FeMnOx-DOM-Cr.

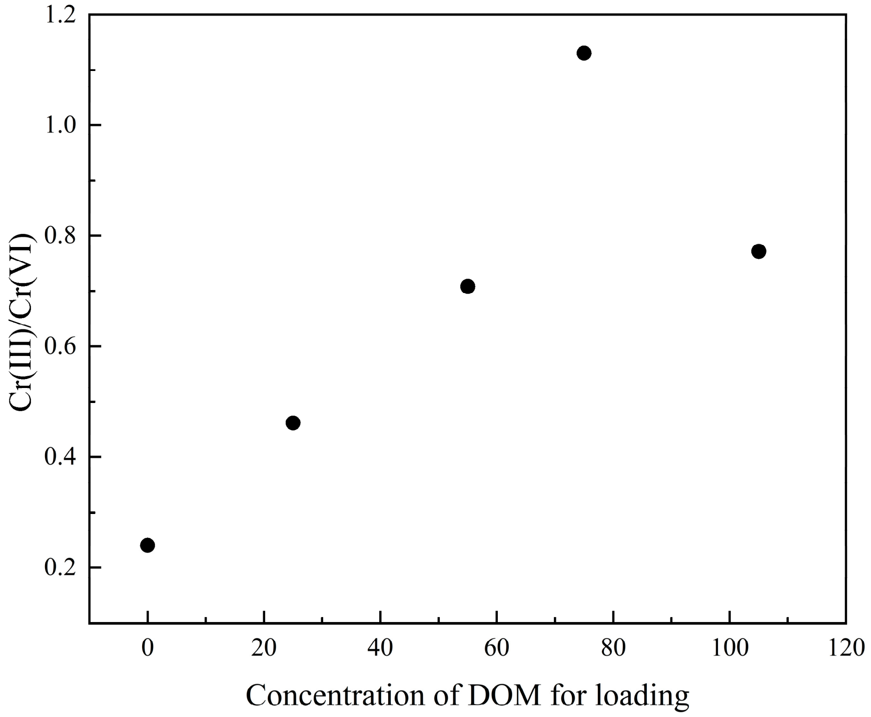

Figure S10 The correlation between DOM loading concentration and Cr(III)/Cr(VI) ratio.
